# Role of imaging in the evaluation of vascular complications after liver transplantation

**DOI:** 10.1186/s13244-019-0759-x

**Published:** 2019-08-14

**Authors:** Juan-José Delgado-Moraleda, Carmen Ballester-Vallés, Luis Marti-Bonmati

**Affiliations:** Medical Imaging Department and Biomedical Imaging Research Group (GIBI230), La Fe Polytechnics and University Hospital and Health Research Institute, Valencia, Spain

**Keywords:** Liver transplantation, Vascular abnormalities, Graft complications, Liver imaging, Doppler ultrasound

## Abstract

Clinical manifestations of liver transplantation complications can be subtle and non-specific. Medical imaging, mainly Doppler ultrasound, plays an important role to detect and grade these. Colour Doppler ultrasound exams are routinely performed at 24–48 h, on the 7th day, the first and third month after transplantation. MDCT and MR images are acquired based on the Doppler ultrasound (DUS) findings, even in the absence of abnormal liver function. As vascular complications appear early after surgery, DUS should be performed by experience personnel. Diagnostic angiography is seldom performed. This pictorial review illustrates the key imaging findings of vascular complications in patients with liver transplantation: hepatic artery complications (such as thrombosis, stenosis of the anastomosis and pseudoaneurysms), portal vein abnormalities (such as occlusion and stenosis) and hepatic veins and/or inferior vena cava flow changes (Budd-Chiari syndrome).

## Teaching Points

- Highlight the importance of early detection of vascular complications after liver transplantation
- Describe key colour Doppler ultrasound findings as the initial imaging test.
- Review the CT and MR imaging findings of the arterial and venous complications.

## Introduction

Currently, liver transplantation is the first-line treatment for patients with terminal liver disease, both acute and chronic.

In recent years, living donor liver transplant has been introduced, especially in children. It reduces the waiting period for a deceased donor transplant and also the ischemic period of the transplanted organ [1]. In this paper, we will focus on cadaveric liver transplantation.

Vascular complications after transplantation are infrequent. Their reported incidence is close to 7% for cadaveric donor liver transplantation and around 13% for living donor liver transplantation [2]. Unfortunately, vascular abnormalities may appear early after surgery, with an associated high incidence of graft loss and mortality [3]. As the clinical manifestations related to vascular injuries are non-specific, early radiological examination plays a major role to make an early diagnosis and establish the best treatment options. Early endovascular treatment is correlated with liver transplantation salvage [4], making early imaging studies especially important.

Colour Doppler ultrasound (CDUS) is the most appropriate imaging test, allowing the early evaluation of the patient, even within the recovery unit after surgery, and also precisely assessing the graft vessels patency [5]. When CDUS shows a vascular abnormality, the surgical anatomy is difficult to interpret or the patient’s clinical status is deteriorating, there is a need to complement the study with either contrast-enhanced CT or MR imaging [6, 7].

Traditionally, surgery has been the first-line treatment of complications, although, nowadays, endovascular treatments have been positioned as first options, limiting surgery to those cases where interventional radiology is limited or failed [8]. Abdominal radiologists should, therefore, foster interventional management when evaluating these patients.

**Fig. 1:**
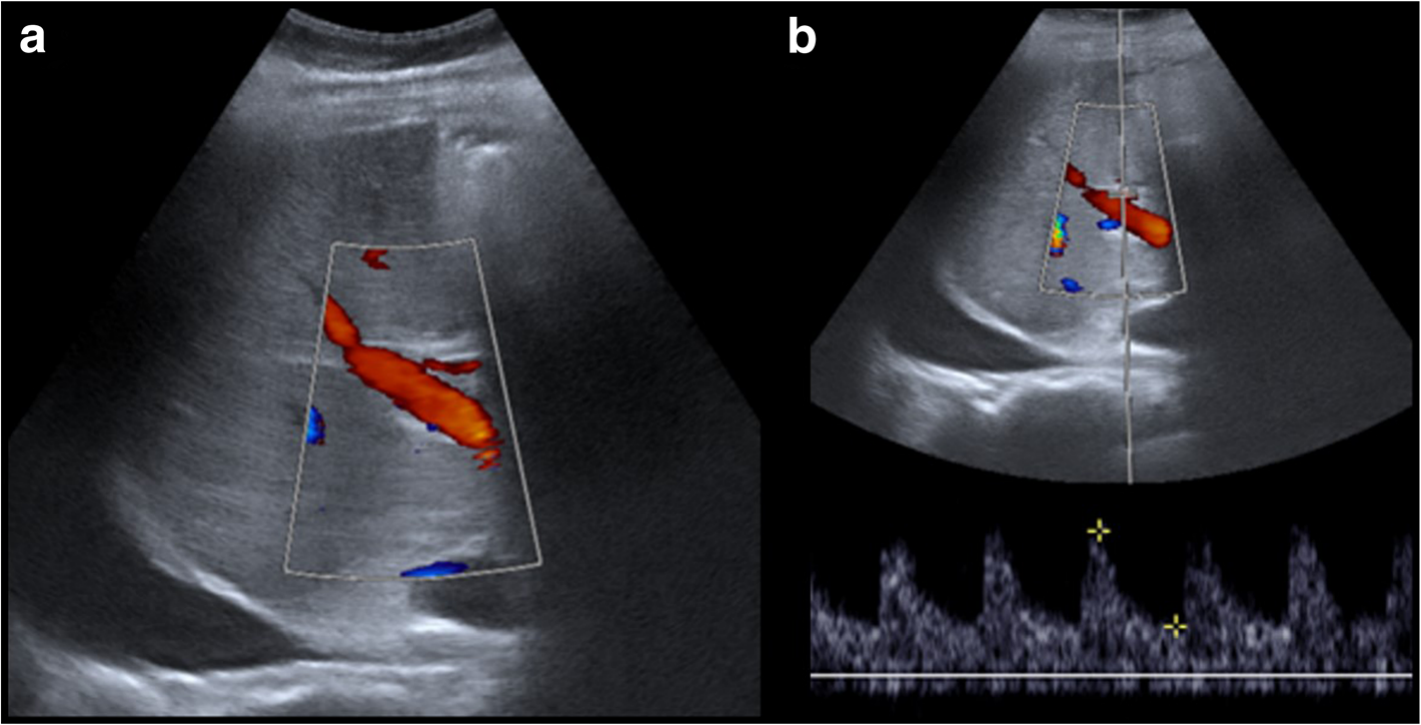
**a**, **b** US control. Postoperative CDUS is performed at 24 h, 48 h, 1 day, and 7 days after surgery. Images show a normal portal and hepatic artery flow

Regarding vascular imaging evaluation, the following protocol is used in our institution, where more than 100 cadaveric liver transplants are performed every year:

- Postoperative CDUS at 24/48 h and day 7 after surgery (Fig. 1). These exams should always be performed [9]. Most patients also have a CDUS at the first and third months.
- Contrast-enhanced CT images are obtained when a vascular lesion is observed on CDUS images or when the liver function is impaired. Occasionally, MR is performed if contraindications to contrast-enhanced CT are present.
- T-tube cholangiography images with direct opacification are also performed on day 4 and the third month to assess the biliary tree.

In recent years, hepatic intra-operative ultrasonography has emerged as a new technique. It allows real-time evaluation of the anastomosis and so immediate treatment before abdominal closure [10].

Usual posttransplantation findings include right-sided pleural effusion, ascites, perihepatic hematoma and periportal oedema. All of them should resolve in the first weeks after surgery [11].

Vascular and biliary anastomoses should be assessed by CDUS. Radiologists should have a clear knowledge of the individual patient postoperative anatomy, since anastomoses are the locations where complications occur most frequently. Moreover, it is important to be aware of the anatomic variants, both in the donor and in the recipient [12].

Currently, the most common surgical technique is orthotopic liver transplantation, where the graft is placed in the right upper quadrant, at the anatomical liver location, after removal of the native liver [13]. Four anastomoses should be carefully assessed: portal vein, bile duct, anastomosis of the recipient inferior vena cava to the donor hepatic veins and anastomosis of the hepatic artery.

The most frequently affected anastomosis is at the hepatic artery [14]. This anastomosis can be made in different places, but the two most frequent are in the hairpin between the right and left hepatic arteries of the recipient or at the outlet of the gastroduodenal artery [15].

## Hepatic artery complications

Related to their onset, hepatic artery complications can be defined as early (within the first month) or late (later than 1 month) complications. Early complications are the most important for patient prognosis because they are associated with graft loss and a high mortality rate.

### Thrombosis (Figs. 2 and 3)

Artery thrombosis is the most serious complication of orthotopic liver transplantation, occurring approximately in 4 to 12% of cases [16]. Thrombosis represents more than 50% of all arterial complications, being the first cause of non-functional liver transplant. Usually, an early complication, it can occur even up to 4 months after transplantation.

**Fig. 2:**
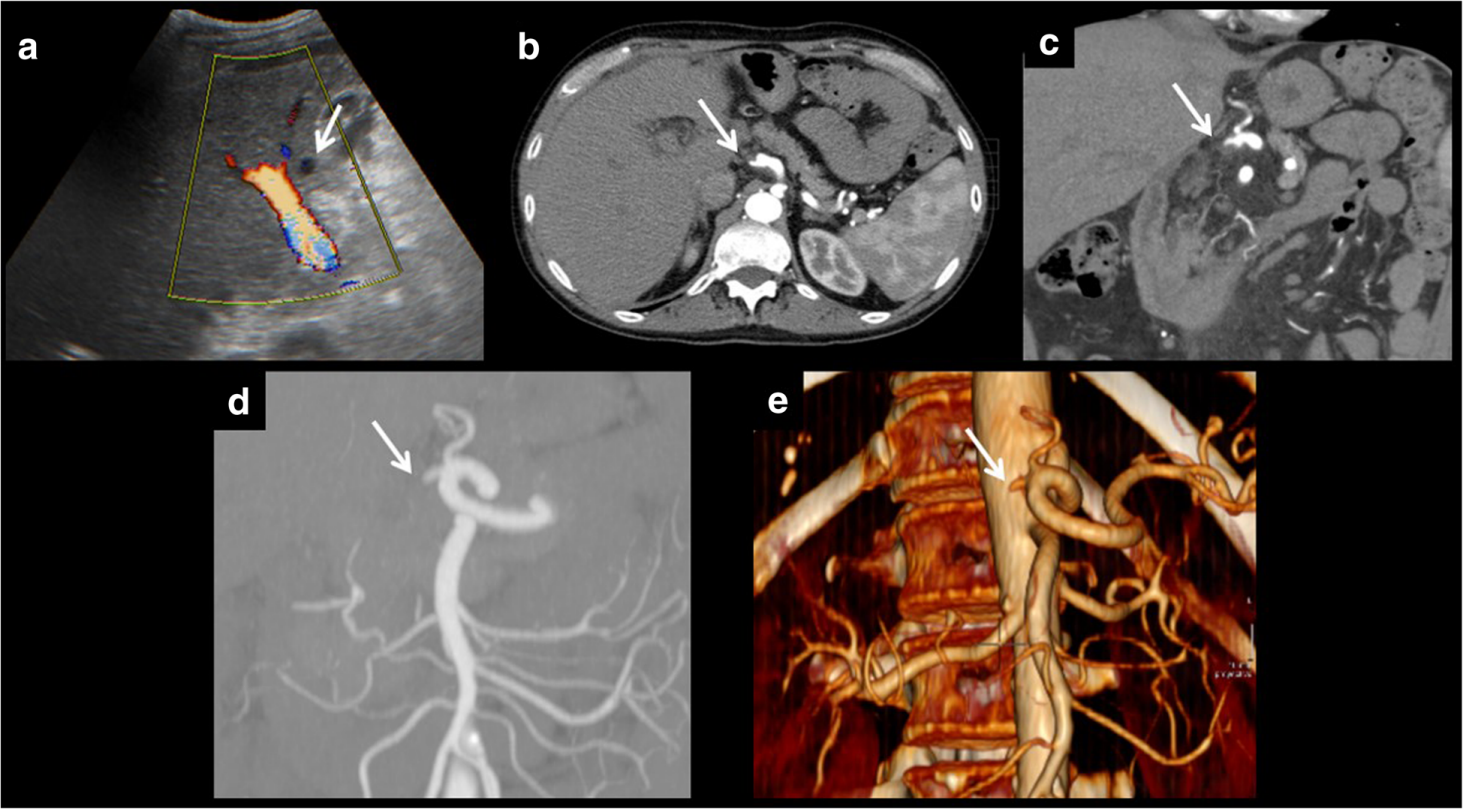
Anastomotic thrombosis. Artery thrombosis is the most serious complication of orthotopic liver transplantation. It can be demonstrated as an absence of flow in Doppler ultrasound examination (**a**). CT can depict the thrombus and also the absence of distal flow (**b**). Multiplanar reconstructions and volume rendering images can be useful to ensure diagnosis (**c**–**e**)

**Fig. 3:**
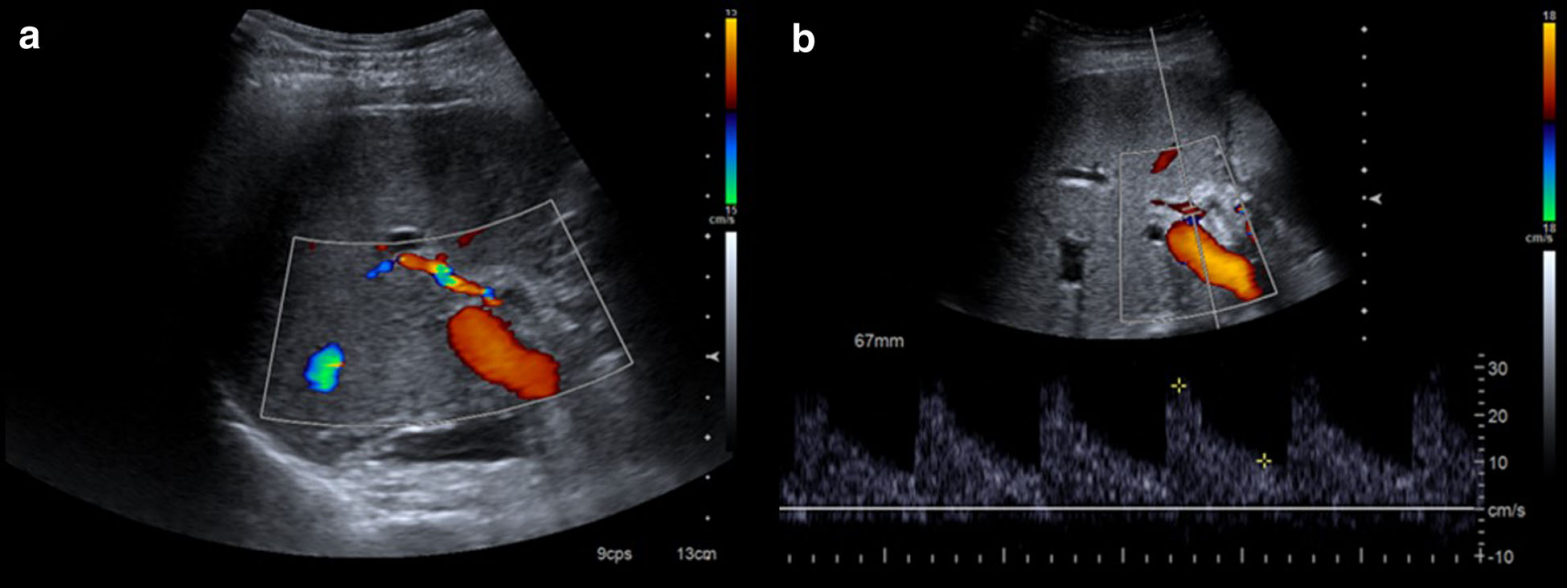
**a**, **b** After treatment control. Control performed after thrombolysis and angioplasty of patient in Fig. 2. Normal arterial flow is seen. Resistive index shows normal values (0.6)

Pulsed and power CDUS has a high sensitivity and specificity to detect the thrombus and grade the degree of stenosis and flow properties, not only at the hepatic artery but also at the intrahepatic branches. Parameters to be considered are hepatic artery diameter, hepatic artery peak systolic velocity and hepatic artery resistance index. Intraoperative ultrasound examination has high sensitivity and specificity for early detection of these findings [17]. Proper quantification of flow velocity within the stenosis by CDUS requires the use of a suitable angle. If the transducer is placed at a parallel or perpendicular angle to the artery whose speed is to be measured, quantified speeds would be lower than actual. Because of the impaired anatomy and generally poor sonographic window, it is usually difficult to find the correct angle. However, resistance index and spectral curve morphology are not affected by angle, being easier to study.

When the blood flow is not identified by CDUS, some different conditions should be considered such as slow flow secondary to vasospasm or low cardiac output. Also, many of these patients have a poor sonographic window due to the surgical dressing materials. Use of a contrast agent (contrast-enhanced US, CEUS) will improve the US diagnostic performance in these cases with a near perfect accuracy [18].

CT angiography is the best technique to further evaluate difficult cases due to its high accuracy, short examination time and facility to be performed with poor patient condition [19]. MRI has proven to have a diagnostic accuracy similar to ultrasound [20], while CT angiography is equivalent [21] or even better [6].

If thrombosis is suspected, a diagnostic arteriography will confirm the diagnosis and allow the best treatment decision. Some endovascular treatments are available to these patients, such as intraarterial thrombolysis (IAT), percutaneous transluminal angioplasty (PTA) and stent placement [8]. If treatment fails, retransplantation should be considered as soon as possible. Studies have shown retransplantation has a better survival rate than endovascular treatment [3]. Nevertheless, because of its less invasive nature, endovascular treatments should be performed as first-line treatment.

### Stenosis (Figs. 4, 5, and 6)

The most frequently affected location is the anastomosis (2 to 13% of patients) [22]. Therefore, this region should me carefully evaluated.

**Fig. 4:**
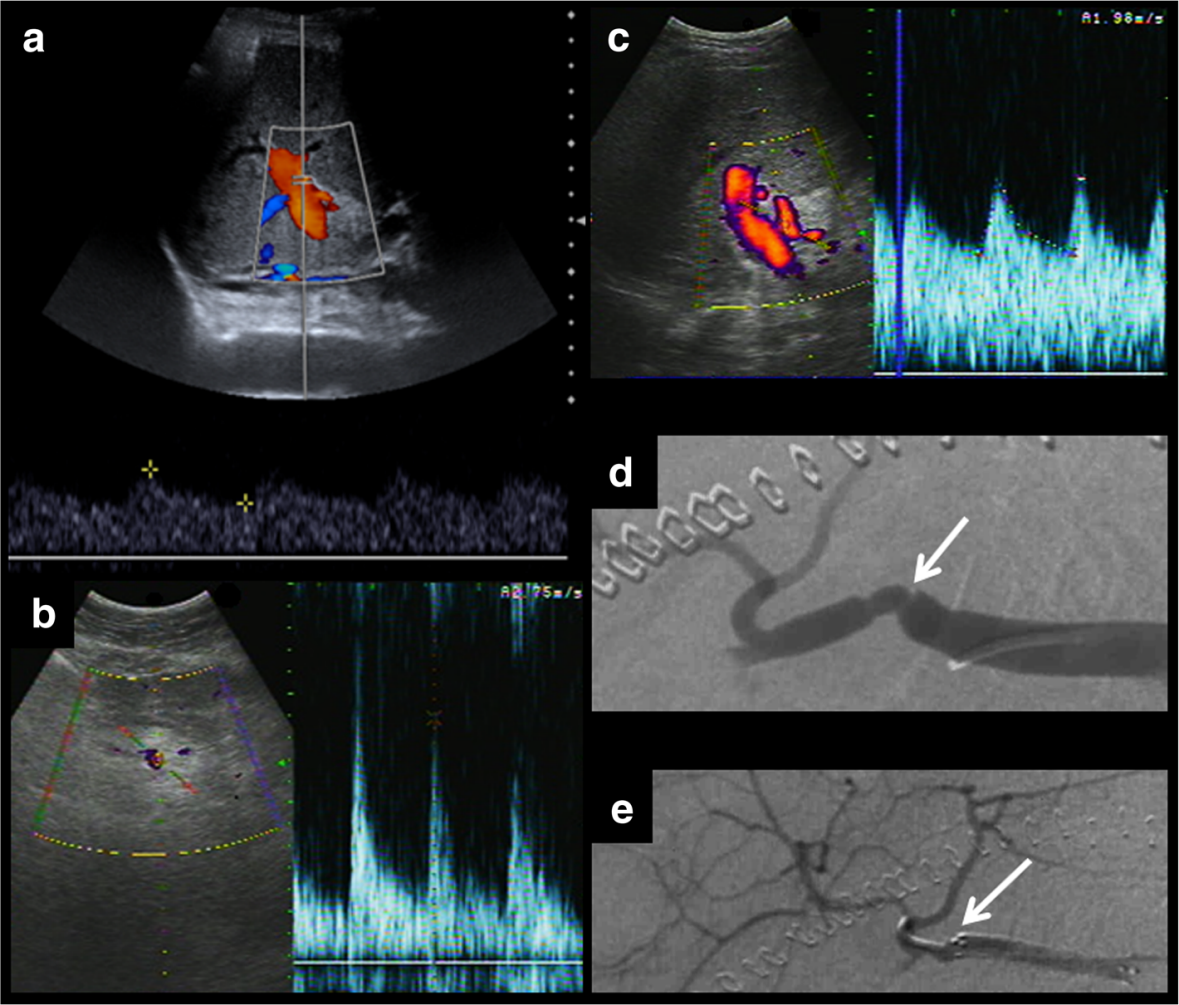
The usefulness of spectral curves on the assessment of hepatic artery stenosis. Even in cases of normal colour Doppler examination (**a**), spectral curves should be obtained. They show a characteristic pattern before and after the stenosis. In the prestenotic segment (**b**), we can appreciate high peaks and an elevated resistive index. In the poststenotic segment (**c**), we can see a parvus et tardus pulse and a low resistive index. Angiography can help confirm the diagnosis (**d**) and perform the treatment (**e**)

**Fig. 5:**
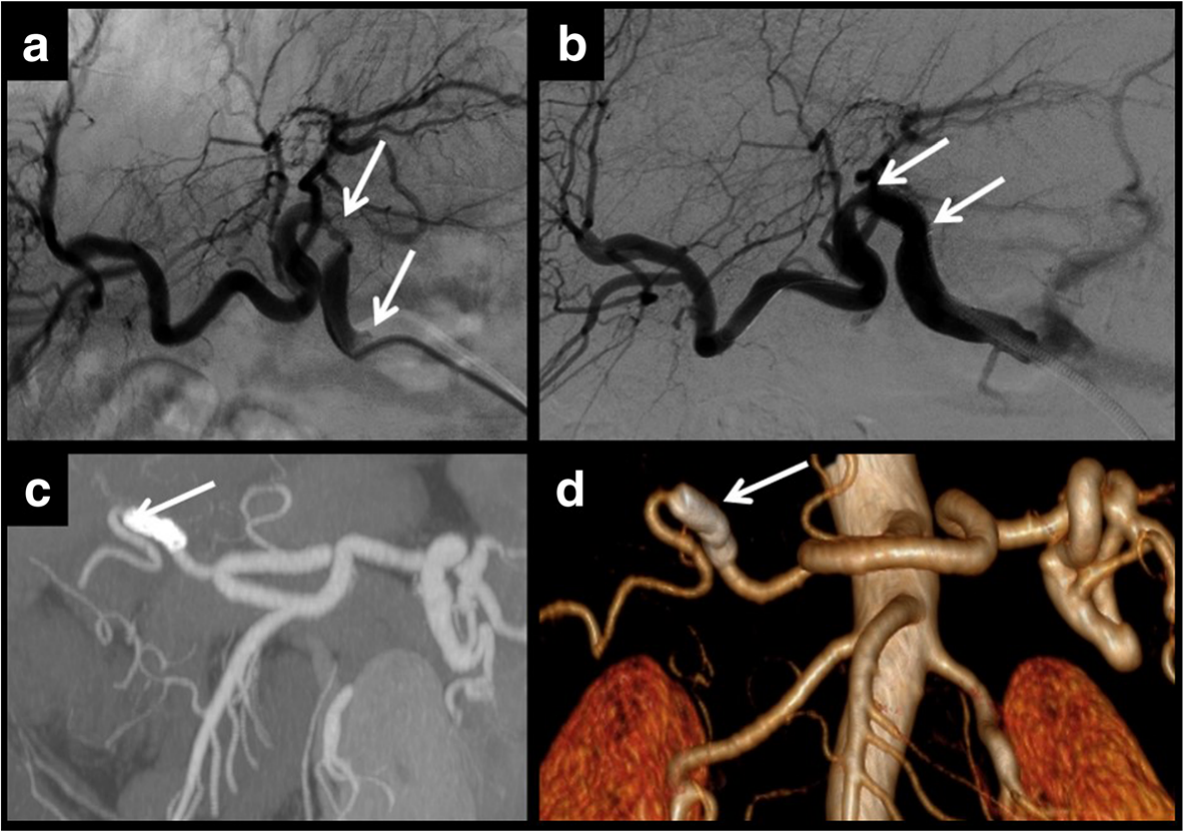
Multifocal stenosis. The hepatic artery can present more than one point of stenosis. In these cases, each one should be treated separately. In this example, angiography demonstrated two points of stenosis (**a**, **b**). Two stents were placed for treatment. Control CT showed that stents had been correctly placed and there is distal artery flow as seen in the MIP reconstruction (**c**) and in volume rendering reconstruction (**d**)

**Fig. 6:**
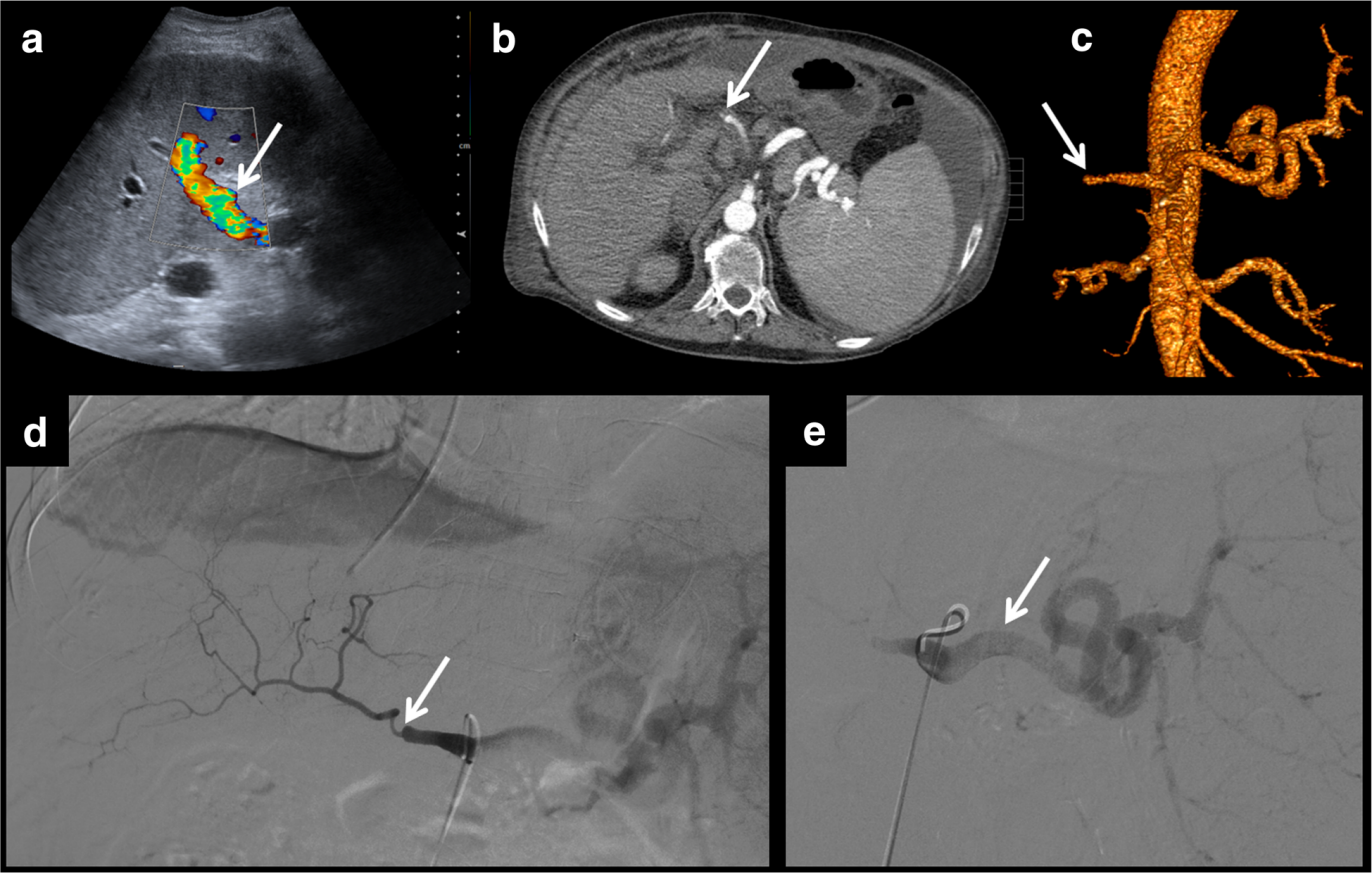
Hepatic artery stenosis leading to splenic artery steal syndrome. Postsurgical US shows a turbulent flow of the hepatic vein. The hepatic artery cannot be clearly identified (**a**). An arterial phase CT is performed, showing severe focal stenosis of the hepatic artery and filiform enhancement of its branches (**b**, **c**). The increased size of the splenic artery should also be noted. Increased splenic artery blood flow explains the increased turbulent portal flow. Angiography confirms both stenosis of the hepatic artery and the increased size of the splenic artery (**d**, **e**). Distal embolisation of the splenic artery was performed as a treatment

Stenosis may progress to thrombosis. So, stenosis and thrombosis are two entities of the same spectrum of vascular complications of liver transplantation.

Stenosis can lead to splenic steal artery syndrome [23].

It usually occurs in the first 3 months (median time to diagnosis 90 to 120 days) [24] [25], but this time shows differences between patients, describing cases that happen even several years after surgery.

Doppler ultrasound is the most useful technique to show this complication, as explained for thrombosis.

It shows a characteristic pattern, depending on the segment studied and its relationship with stenosis. Power Doppler is also useful to quantify blood flow and study spectral curves [26].

- Prestenotic segment shows elevation of resistance index (more than 0.8) and low flow.
- Stenotic segment displays a very high flow rate and aliasing artefact, due to the turbulent flow. Blood systolic peak is more than 200 cm/s.
- Poststenotic segment presents a low resistance index (less than 0.5) and a parvus et tardus morphology of the spectral curves with long systolic acceleration time (more than 0.08 s). It has also a turbulent flow.

#### An important consideration

It is important to remember that, in the first 3 days after liver transplantation, an increased resistance index of the hepatic artery (greater than 0.8) is found in approximately 50% of the patients [27].

If found, it should be monitored until it has normalised, typically in the fourth day after the transplantation [28].

Although the severity of the described findings correlates with the degree of the stenosis, ultrasound does not allow proper quantification of this: the technique of choice is CT angiography [29]. In addition, CT allows proper evaluation of patients with a poor sonographic window. Multiplanar and three-dimensional curved reformatting are useful to measure the vessel lumen.

MRI angiography is a limited technique because of a relatively high false-positive rate [20].

Hepatic artery stenosis requires early treatment. First, an angiography and an angioplasty should be made. If this procedure fails, surgery is required. Once again, retransplantation has a better outcome, but is preferred as a second-line treatment, to use when endovascular therapy does not work [3].

### False aneurysm or pseudoaneurysm (Figs. 7 and 8)

Pseudoaneurysm of the hepatic artery and its branches presents the same features as in other parts of the body, differently to other vascular complications of liver transplantation. In this case, it can affect any of the branches, not only the site of the anastomosis.

**Fig. 7:**
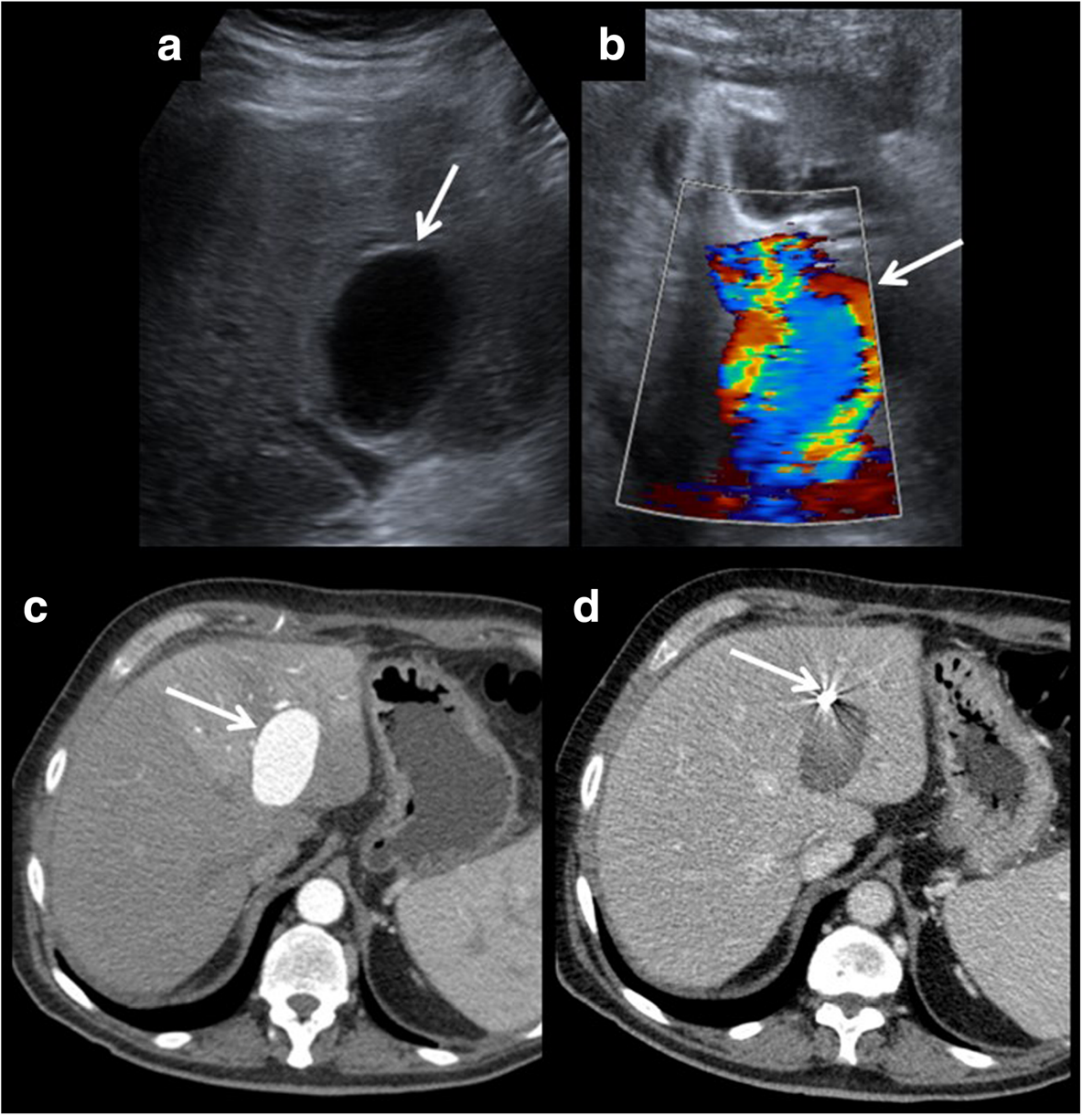
Hepatic artery pseudoaneurysm. Pseudoaneurysms can be discovered using ultrasound (**a**). It shows a characteristics appearance in Doppler ultrasound, due to the turbulent forward and backward flow (**b**). Arterial phase CT shows arterial enhancement of the pseudoaneurysm (**c**). A coil is placed to block entering the blood and to prevent rupture (**d**).

**Fig. 8:**
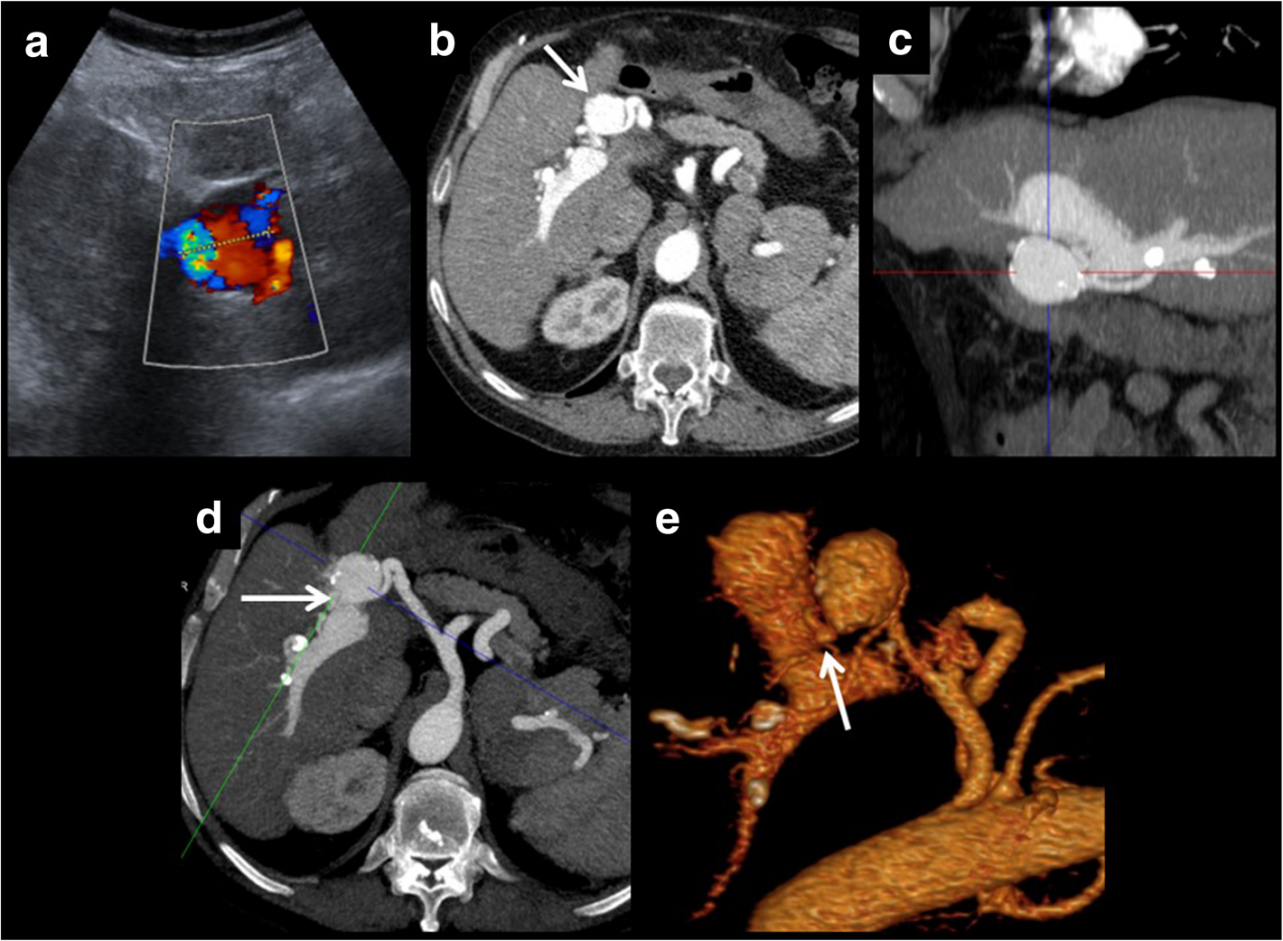
Intrahepatic artery pseudoaneurysm complicated with portal fistula. On these images, an intrahepatic false aneurysm is presented. It shows turbulent flow on colour Doppler examination (**a**) and arterial enhancement on CT (**b**). One of the possible complications of this entity is the development of portal or biliary fistulas. In this case, a porta fistula is seen (portal branches show arterial enhancement). Multiplanar and volumetric reconstructions help to find the exact location of the fistula (**c**–**e**).

It is a rare complication, with an incidence of 2.5% of the cases of liver transplantation, according to the retrospective study having the largest sample. It has no bias for any of the indications of liver transplantation [30].

In ultrasound, pseudoaneurysms present as a hypoechoic structure with turbulent blood flow within as observed by colour Doppler. Typically, due to swirl formed by the inlet and blood outlet, it is possible to observe the yin-yang sign [31].

If CT angiography is performed, it characteristically presents an arterial enhancement equal to the other arterial vessels, with an equal wash out in the later stages.

They are classified according to their location: extrahepatic and intrahepatic.

#### Extrahepatic pseudoaneurysms

The most common site is the arterial anastomosis. They can occur spontaneously or as a complication of treatment of a preexisting stenosis. Bacterial or fungal infection isolated from the peritoneal fluid or from the pseudoaneurysm wall can be present in up to 81% of cases, according to series [30].

#### Intrahepatic pseudoaneurysms

Characteristically, it is a complication of percutaneous liver biopsy, the usual cause, but also may be secondary to bile duct infections [5]. In case of rupture of such aneurysms, a portal or biliary fistula can appear. Pseudoaneurysms and fistulas secondary to percutaneous liver biopsies are much more frequent if the biopsy is done in the first days or weeks after transplantation. In fact, according to some reports, the risk of developing an arterioportal fistula is approximately 50% for biopsies performed in the first week, dropping to 10% if performed approximately 1 month after surgery [29].

In both cases, treatment consists of coil embolisation and stent placement to prevent inflow to the pseudoaneurysm. If the results of this treatment are not satisfactory, surgical resection can be performed [32].

### Ischaemia/liver infarction

Hepatic infarction is very rare in normal patients, since the liver is a richly vascularised organ with blood from different circuits: the hepatic artery and the portal vein. Inside the liver, there are numerous anastomotic vessels and collateral branches.

However, in liver recipient patients, anastomoses are stopped, so hepatic infarction is much more common. It is usually associated with arterial occlusion (85% of cases), and, rarely with portal vein occlusion [29]. Bile ducts are especially sensitive to arterial blood flow impairment because they receive all their blood supply from the hepatic artery [33].

Ischaemia and liver infarction can be consequences of all three described alterations of the hepatic artery: thrombosis, stenosis and pseudoaneurysm.

## Complications of the portal vein

Portal vein complications are infrequent. They affect less than 2% of liver transplantations [34].

The most common surgical technique is to directly anastomose the portal vein of the donor with that of the recipient.

However, sometimes this is not possible, because there is a portal thrombus which prevents direct anastomosis. In such cases, it is necessary to remove the segment occupied by the thrombus and perform a bypass. Typically, the donor iliac vein is used to make the bypass [35].

Complications of the portal vein usually affect the anastomosis, so it is important to know its location.

The most common complications of the portal vein will be explained.

### Thrombosis (Fig. 9)

It is a rare complication. It occurs most often in the extrahepatic portal vein, at the site of the anastomosis. It can be proved by the absence of Doppler flow on ultrasound examination or a filling defect in the contrast-enhanced CT scan or MRI.

**Fig. 9:**
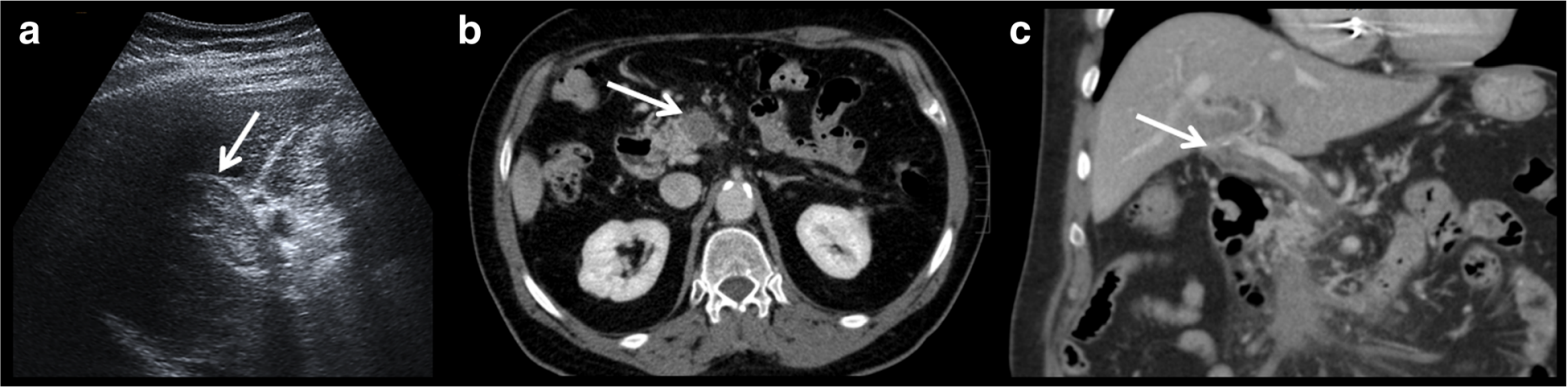
Portal vein thrombosis. Echogenic material is observed on ultrasound examination (**a**). This finding is confirmed in axial CT (**b**). Multiplanar reconstructions help in the assessment of the extension (**c**)

Some studies show that contrast-enhanced ultrasound is a promising technique being able to identify cases of thrombosis missed by other imaging techniques [36].

If thrombosis is seen in the first 72 h after surgery, surgical revision of the anastomosis should be made. Later, the treatment is done with percutaneous thrombolysis, angioplasty, stenting or, if unable to perform the treatment with these less invasive techniques, surgery.

### Stenosis (Figs. 10 and 11)

The site most frequently involved is the anastomosis. The characteristic findings in each imaging technique are as follows:

- Doppler ultrasound, there is typically an increase in portal blood flow velocity at the point of the anastomosis (greater than 125 cm/s) or three times higher at the site of the stenosis in comparison with the prestenotic segment [37]. As explained for arterial velocity, Doppler measurements require using a correct angle, which is not always possible to obtain because of patient conditions. Consequently, the stenotic/prestenotic ratio is a more accurate measurement.
  An aliasing artefact can also appear in the stenotic segment because of turbulent flow. This can be also a normal finding in the early postoperative period. So, in the case of turbulent flow, anastomosis should be evaluated in future controls, for comparison [5].
- CT or MR angiography can observe and quantify the degree of stenosis.

**Fig. 10:**
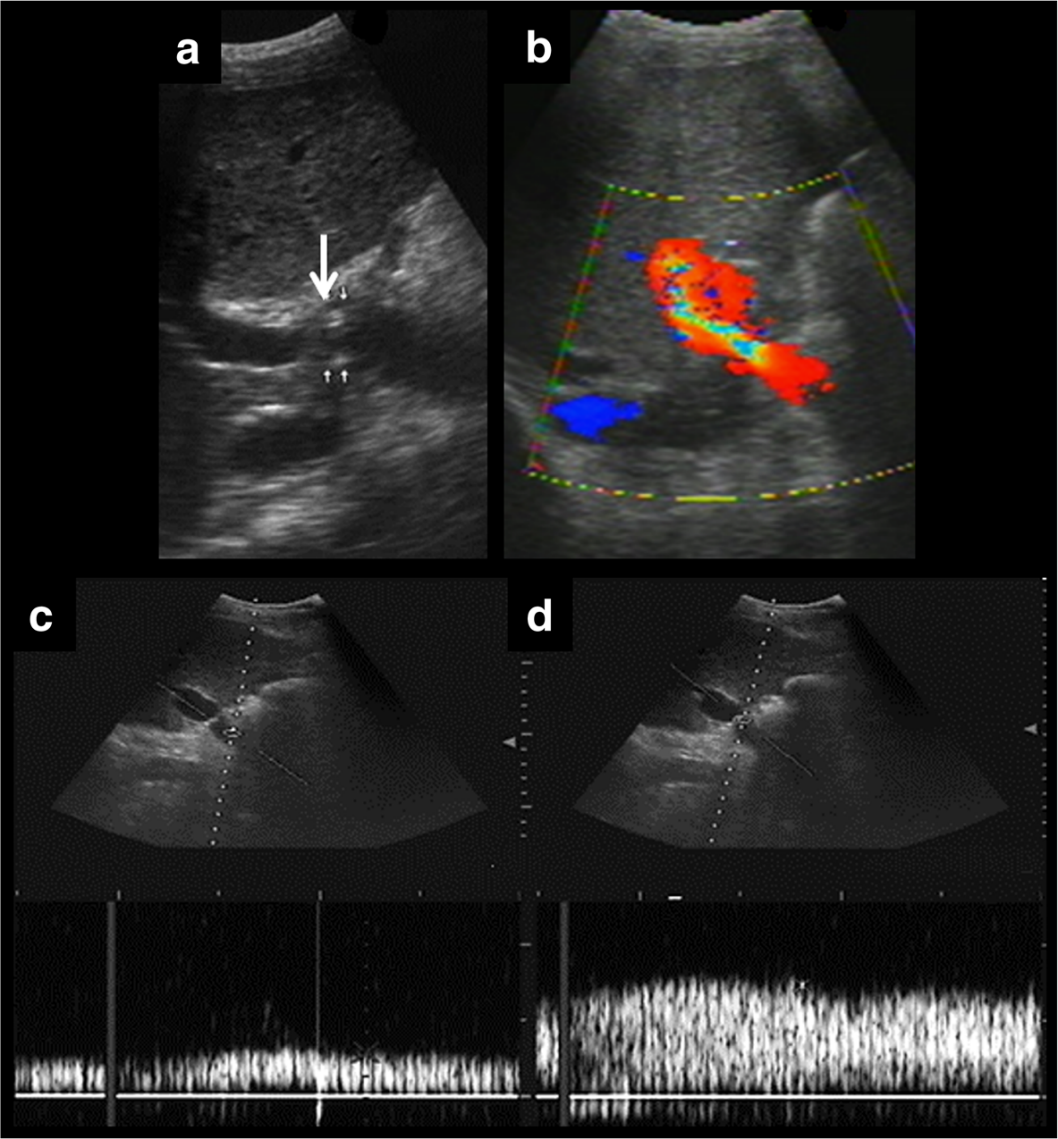
Usefulness of spectral curves on the assessment of portal vein stenosis. Ultrasound examination shows multiple points of stenosis (**a**). Colour Doppler demonstrates turbulent flow (**b**). Spectral curves show a characteristic pattern before and at the stenosis. In the prestenotic segment (**c**), we can appreciate a low flow. In the stenotic segment (**d**), high-speed flow is seen (peak velocity over 125 cm/s). Stenotic/prestenotic ratio is a useful measurement. In this case, the result is clearly more than three

**Fig. 11:**
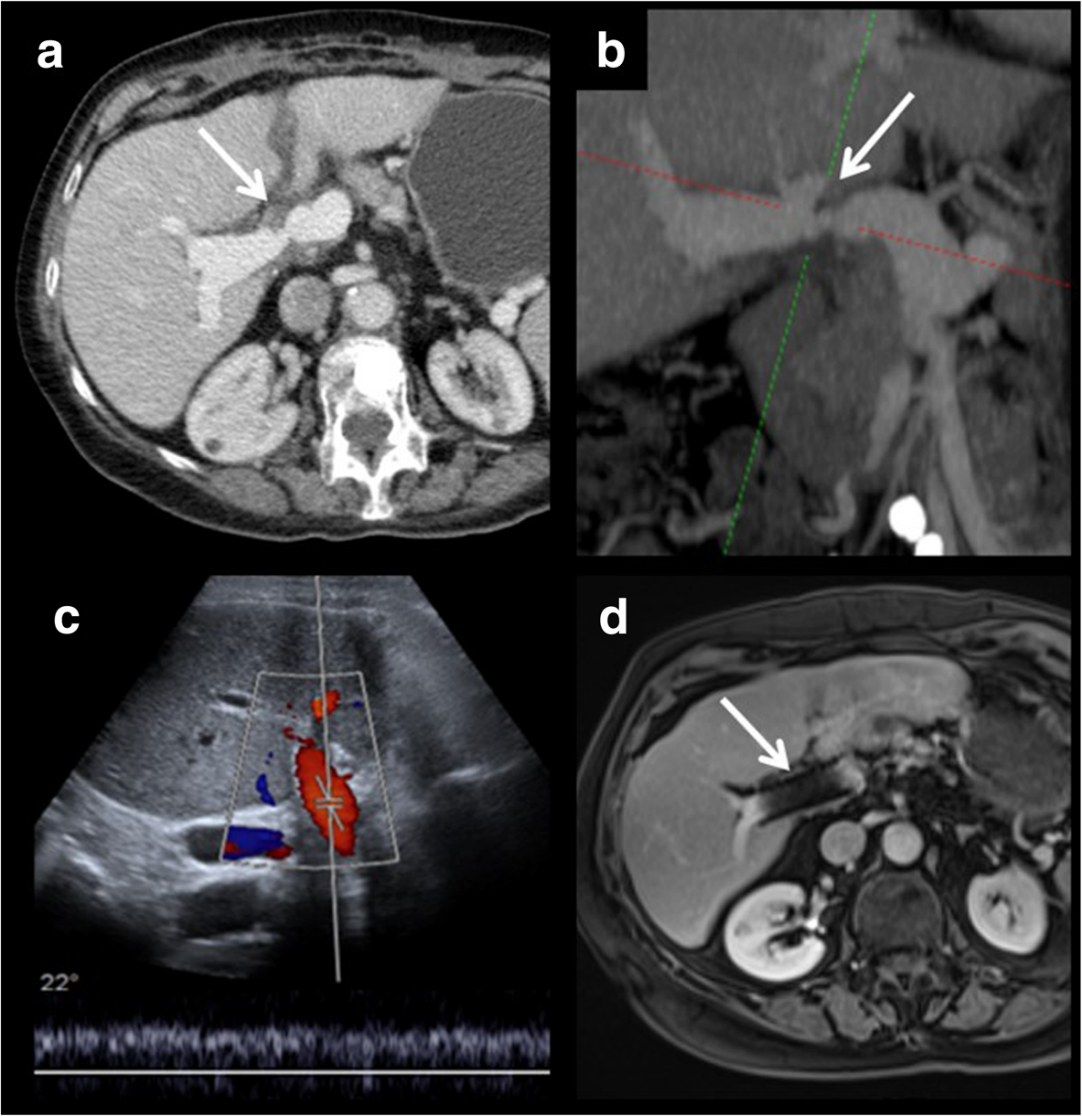
Portal vein stenosis. Portal vein stenosis is demonstrated on CT, both on axial images (**a**) and in multiplanar reconstructions (**b**). After stent placement, a control ultrasound is performed (**c**). It shows normalisation of the portal flow. Control MRI (**d**) shows metallic artefact of the stent. Normal contrast enhancement is seen both proximal and distal to the stent

Stenosis should be carefully differentiated from a physiological mild reduction in vessel calibre at the anastomotic site. This finding in more appreciable if there is a size discrepancy between the donor and recipient portal veins. In this case, the focal narrowing is a normal finding and it is not related to stenosis. Knowledge of preoperative anatomy and assessment of the graft will help to make the differential diagnosis. Nevertheless, this finding should be followed-up, because it predisposes to the development of a stenosis [35]. Treatments are angioplasty, stenting and, in case of failure of the prior techniques, surgical resection.

### Ischaemia/liver infarction

Although much more common in the case of arterial complications, it may also occur as a result of stenosis or portal vein thrombosis.

## Complications of the inferior vena cava

Inferior vena cava complications are in frequent. They affect less than 2% of liver transplantations [34]. Coronal reconstructions are especially useful in measuring the extension of the thrombus.

### Thrombosis (Fig. 12)

Usually, they occur because of the surgical technique or a hypercoagulable state. Diagnosis and treatment are similar to that of portal vein thrombosis.

**Fig. 12:**
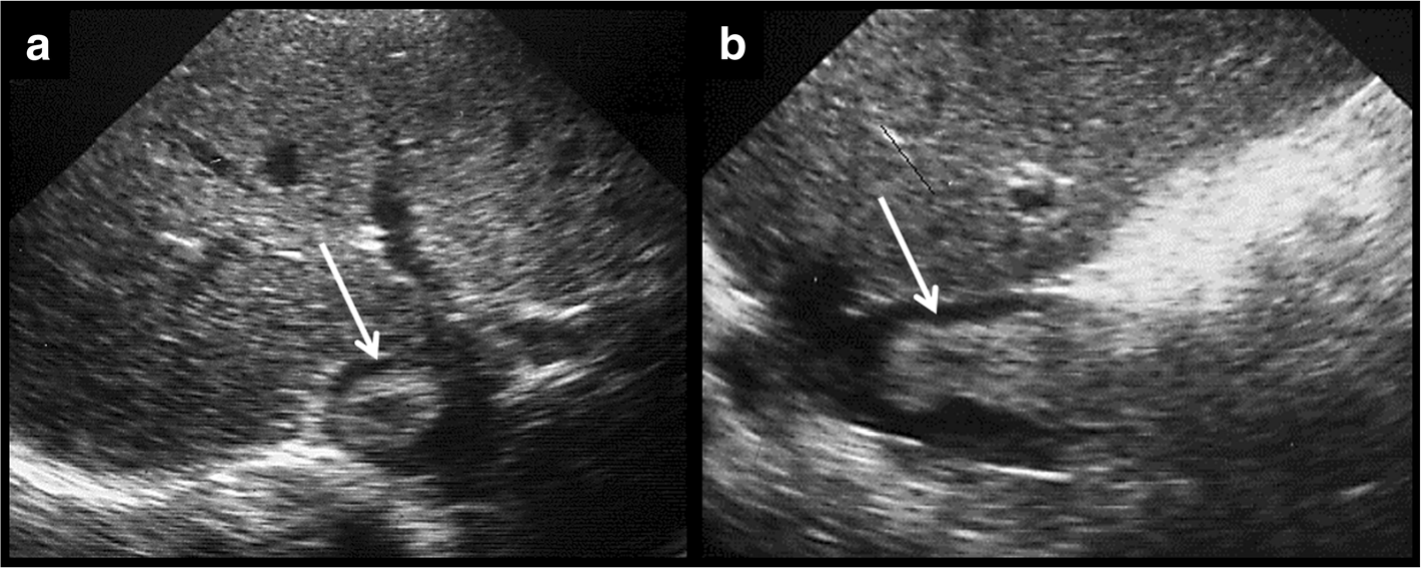
**a**, **b** Thrombosis of the inferior vena cava. Thrombosis of the inferior vena cava was discovered through an axial ultrasound examination. Sagittal examination helps to asses its extension.

### Stenosis (Fig. 13)

Just as in the other vessels, the most common site is the anastomosis. It is also possible that extrinsic compression stenosis occurs, secondary to oedema of the graft or fluid collections, hematomas or abscesses. The diagnostic and therapeutic techniques are similar to those described in the portal vein.

**Fig. 13:**
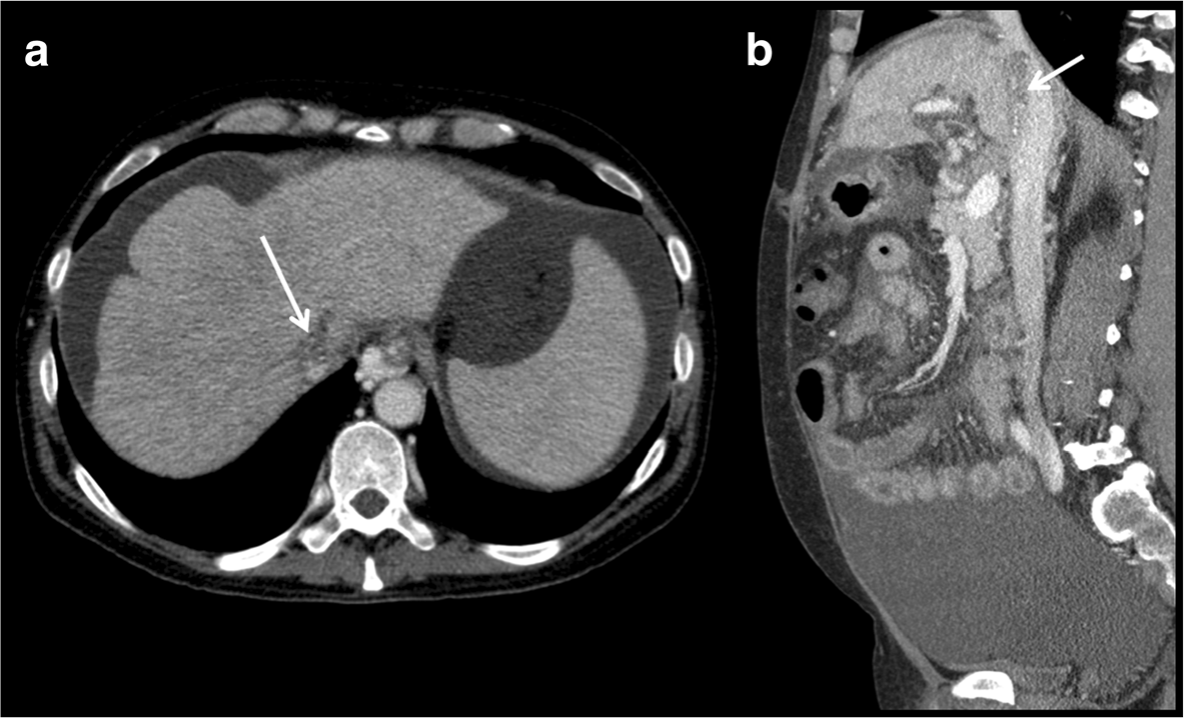
**a**, **b** Stenosis of the inferior vena cava. Stenosis of the inferior vena cava is seen on the axial and sagittal images. It affects hepatic vein confluence. Difficulty with venous drainage produces ascites. Percutaneous angioplasty is performed for treatment

## Complications of hepatic veins (Fig. 14)

These are rare complications. As in the rest of the vessels, characteristic complications are thrombosis (Budd-Chiari syndrome) and stenosis.

**Fig. 14:**
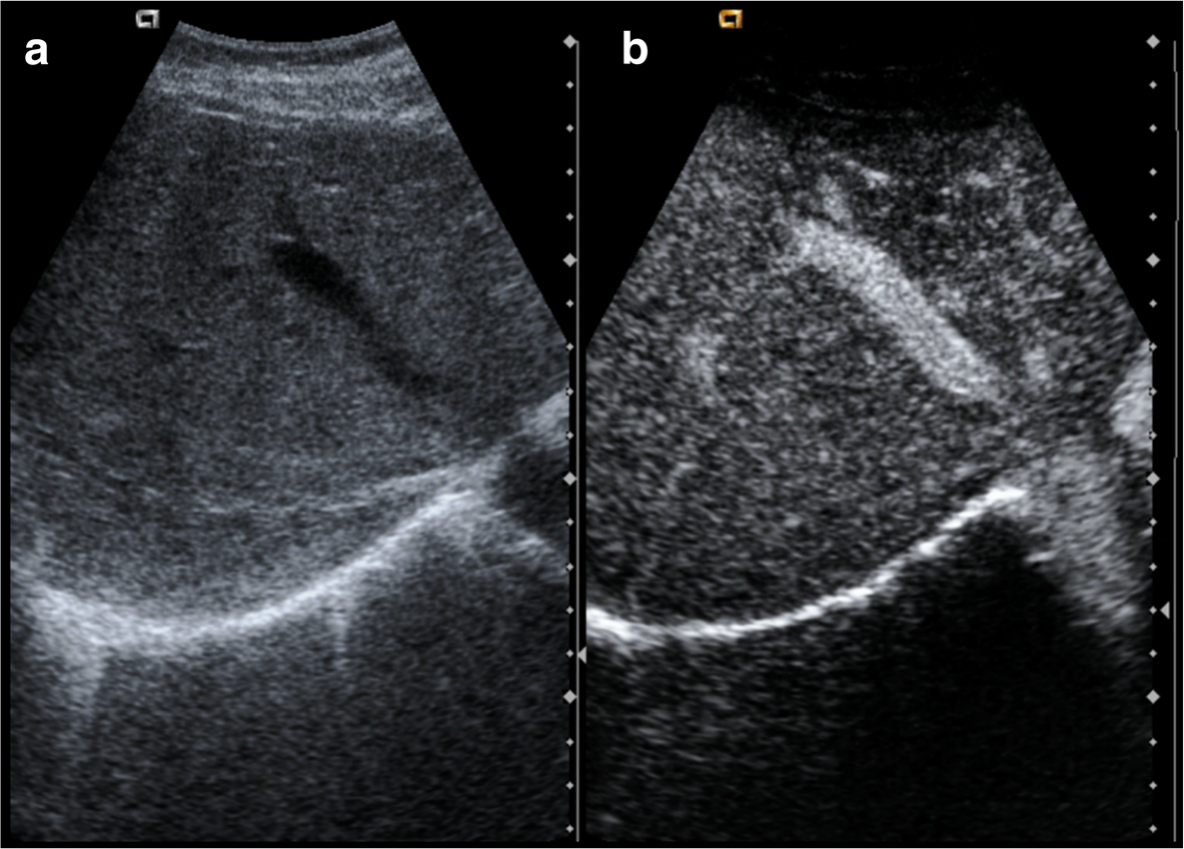
**a**, **b** Thrombosis of the right hepatic vein. The right suprahepatic vein is more echogenic than the middle hepatic vein. After contrast administration, there is an enhancement of the middle hepatic vein, showing normal flow. The right hepatic vein does not enhance because of thrombosis

In the case of living donor transplantation, knowledge of the surgical anatomy is very important. Usually, the right hepatic vein is preserved in the right lobe of the graft, but the middle hepatic vein is usually left for the safety of the donor. Sometimes, the middle hepatic vein is also included in the graft, and therefore, it should be assessed in the postoperative control [38].

## Conclusion

Early detection of vascular complications of liver transplantation is essential in establishing effective treatment: this determines the effectiveness of transplantation and patient mortality and morbidity.

Doppler ultrasound is the initial imaging test. If it does not allow definitive diagnosis, other techniques (contrast-enhanced ultrasound, CT, MRI) are indicated.

## Data Availability

Data sharing is not applicable to this article as no datasets were generated or analysed during the current study.
